# Extraribosomal Functions of Cytosolic Ribosomal Proteins in Plants

**DOI:** 10.3389/fpls.2021.607157

**Published:** 2021-04-21

**Authors:** Wei Xiong, Ting Lan, Beixin Mo

**Affiliations:** ^1^Guangdong Provincial Key Laboratory for Plant Epigenetics, Longhua Bioindustry and Innovation Research Institute, College of Life Sciences and Oceanography, Shenzhen University, Shenzhen, China; ^2^Key Laboratory of Optoelectronic Devices and Systems of Ministry of Education and Guangdong Province, College of Optoelectronic Engineering, Shenzhen University, Shenzhen, China

**Keywords:** ribosome, RP genes, plant ribosomal protein, extraribosomal functions, gene duplication

## Abstract

Ribosomes are basic translational machines in all living cells. The plant cytosolic ribosome is composed of four rRNAs and approximately 81 ribosomal proteins (RPs). In addition to the fundamental functions of RPs in the messenger RNA decoding process as well as in polypeptide synthesis and ribosome assembly, extraribosomal functions of RPs that occur in the absence of the ribosome have been proposed and studied with respect to RPs’ ability to interact with RNAs and non-ribosomal proteins. In a few cases, extraribosomal functions of several RPs have been demonstrated with solid evidences in plants, including microRNA biogenesis, anti-virus defenses, and plant immunity, which have fascinated biologists. We believe that the widespread duplication of RP genes in plants may increase the potential of extraribosomal functions of RPs and more extraribosomal functions of plant RPs will be discovered in the future. In this article we review the current knowledge concerning the extraribosomal functions of RPs in plants and described the prospects for future research in this fascinating area.

## Introduction

Ribosomes have many universal features, but ribosomes of each specific organism also have their own unique properties. The cytosolic 80S ribosomes in eukaryotes are composed of the 60S large subunit and the 40S small subunit. The plant 60S large subunit contains 25S, 5.8S, and 5S rRNAs and 48 RPLs (Ribosomal Protein of the Large subunit), whereas the 40S small subunit contains 18S rRNA and 33 RPSs (Ribosomal Protein of the Small subunit) (Chang et al., 2005). rRNAs are proved to catalyze the key steps in protein synthesis. RPs play essential roles in mRNA recognition and decoding, tRNA recognition, subunit assembly, transport, and stabilization (Ban et al., 2000; Klein et al., 2004). Interestingly, free RPs have been reported to act outside of the ribosome in rRNA processing and folding in yeast (Ferreiracerca et al., 2007; Ross et al., 2007), although these functions are still related to ribosome biogenesis.

In addition to these canonical roles, emerging evidences have demonstrated that RPs may have widespread extraribosomal functions in transcription, the cell cycle, mRNA processing, DNA repair, apoptosis and tumorigenesis (Naora and Naora, 1999; Weisberg, 2008; Lindstrom, 2009; Warner and Mcintosh, 2009; Molavi et al., 2019; Zhao et al., 2019). The first discovered extraribosomal function of RPs was that of free *E. coli* RPS1, which could bind to specific sites on the genome of the bacteriophage Qβ. RPS1 together with three other proteins serve as the RNA replicase, which is responsible for the replication of the bacteriophage’s genome (Blumenthal and Carmichael, 1979). Years later, several extraribosomal functions of RPs were demonstrated in different types of organisms. For example, RPS3 from *Drosophila* or mammalian cells was observed to have the ability to nick DNA at basic sites (Wilson et al., 1994); the Archaeal RPL7 was demonstrated to function as an sRNP core protein that binds the box C/D snoRNA core motif (Kuhn et al., 2002), in mammalian cells, many different kinds of stress could cause accumulation of ribosome-independent free RPs, which could perform extraribosomal functions involved in tumorigenesis or immune response by participating in different signaling pathways (Zhou et al., 2015; Bohnsack and Bohnsack, 2019).

As mentioned above, over the past decade, numerous advances have been made in revealing the biochemical, cellular, and physiological roles of RPs beyond the ribosome in prokaryotes and eukaryotes. The biological functions of RPs in plants are even more diverse, and ribosomes are heterogeneous because RP genes are highly duplicated and each RP gene has two or more copies in plants (Barakat et al., 2001; Whittle and Krochko, 2009). Duplicated RP genes may undergo functional specialization or may acquire new functions (Lynch and Conery, 2000).

A mutation in a given single RP in plants can not only affect the functions of the RP itself, but can also influence the properties of the ribosome, causing ribosome insufficiency or the partial dysfunction of the ribosome, which could bring changes in translation in varying degrees (Horiguchi et al., 2011). Several different *rp* mutants were reported to share common phenotypes: pointed leaves, short roots, delayed flowing time, aberrant venation patterns and reduced fertility. Ribosomal profile was performed to evaluate the consequence on translation resulted from a RP mutation. Interestingly, the ribosomal profile of two different *rp* mutants *rpl5a* and *rpl4d*, both of which exhibit severe developmental defects, only have minor differences compared to wild type, suggesting the general translation state of most mRNAs in *rpl5a* and *rpl4d* is unaltered. However, specific mRNAs with upstream ORFs in their 5’-leader sequences are translated with extremely low efficiency in *rpl5a* and *rpl4d* (Rosado et al., 2012). Similar developmental defects caused by mutations in different RP genes strongly indicate that ribosomes are important players controlling developmental processes. On the other hand, some *rp* mutants have specific phenotypes which are not observed in mutants of other RP families. For example, *rps27b* was reported to be more sensitive to UV irradiation and methyl methane sulphate (MMS) than wild type (Revenkova et al., 1999). Mutations in these RPs still influence the integrity of ribosomes in structure and function, but it is also possible that these specific phenotypes are caused by the loss of extraribosomal functions of these RPs.

In the past few years, several plant RPs have been demonstrated to function outside of the ribosome in several important physiological activities including microRNA biogenesis, anti-virus defenses, plant immunity. Here, we highlight the emerging concepts about the extraribosomal functions of RPs and summarize new developments in this area in plants.

## The Concept of the Extraribosomal Functions of Rps

Most RPs are basic, with a pI range from 8.1 (RPS27) to 12.8 (RPS30 and RPL39); however, RPSa, RPP0, RPP1, RPP2, RPP3, and RPS12 are acidic (pI: 4.0–5.8) in *Arabidopsis* (Barakat et al., 2001). The positive charges of the majority of the RPs are in accordance with their association with rRNAs and their abilities to decode mRNA and recognize tRNA during translation (Brodersen and Nissen, 2005). Interestingly, numbers of RPs were reported to have abilities to bind other types of RNAs, DNAs, and non-ribosomal proteins. For example, RPS27, RPS29, and RPL10 were proved to contain the conserved zinc finger motif which could undertake a wide variety of cellular roles by supplying stable structural scaffolds and promoting crucial binding interactions, especially among RNAs, DNAs, and proteins (Chan et al., 1993; Imafuku et al., 1999; Isalan, 2020). In the past few decades, many extraribosomal functions of RPs have been discovered in different types of organisms (Warner and Mcintosh, 2009). Additionally, several findings have suggested that extraribosomal functions of RPs may exist, although the exact mechanism is not yet clear.

The evolutionary derivation of the extraribosomal functions of RPs remains unclear. rRNA was thought to be the main, and perhaps the only, catalyst in the ribosome, and it is widely believed that the earliest protein-synthesizing machines were exclusively composed of rRNA (Wool, 1996; Green and Noller, 1997; Zimmermann, 2003; Moore and Steitz, 2011). It is unknown as to whether RPs coevolved with rRNA to specifically enhance the properties of ribosomes or whether RPs were later recruited from other cellular systems to augment the speed and fidelity of protein synthesis. If the latter case is true, the existence of extraribosomal functions of RPs may have originated prior to the appearance of their roles in protein synthesis.

Researchers had suggested that there are three criteria to determine whether an RP performs extraribosomal function: (1) the RP specifically interacts with non-ribosomal components in the cell (presumably RNA, protein or even DNA); (2) such an interaction has a physiological effect on a living (or dying) cell; and (3) there is evidence that the activity is occurring at a distance away from the ribosome (Wool, 1996; Naora and Naora, 1999; Lindstrom, 2009; Warner and Mcintosh, 2009). These three criteria for evaluation of extraribosomal functions of RPs are very stringent. In some cases, there are substantial evidences supporting the extraribosomal functions of RPs, whereas in most cases the evidences are not solid.

## RP Genes Are Highly Duplicated in Plants Compared to Other Organisms

The organization of the RP genes is diversified in different organisms. Each RP in plants is encoded by a gene family comprised of two or more highly homologous copies resulting in highly heterogeneous ribosomes in plants (Barakat et al., 2001; Giavalisco et al., 2005; Whittle and Krochko, 2009; Savada and Bonhamsmith, 2014). Duplicated plant RP genes within the same family may lose their function (Pseudogenization), may share their ancestral gene function (Subfunctionalization), or may acquire new functions (Neofunctionalization) (Lynch and Conery, 2000). For instance, in *Arabidopsis thaliana*, RPS5 family contains two members, RPS5A was proved to be expressed in rapidly dividing cells in early embryonic development, whereas its paralog RPS5B was reported to be expressed in cells undergoing differentiation (Weijers et al., 2001). And there are growing evidences supporting that functional specialization, expression pattern divergence, and subcellular localization difference exist among different paralogs within the same ribosomal protein family (Whittle and Krochko, 2009; Savada and Bonhamsmith, 2014). Thus, the RPs in plants may exhibit considerable potential for acquiring extraribosomal functions.

## Extraribosomal Functions of RP Genes in Plants

### Free RPL10A Involves in Transcriptional Regulations of Certain Genes

RPL10 is a component of the large subunit of the cytosolic ribosome. Based on the cryo-electron microscopy (cryo-EM) analysis results, RPL10 is located on the inter-subunit side of the large ribosomal subunit (Natchiar et al., 2017). RPL10 is required for the 60S large ribosomal subunit biogenesis, nuclear export, 40s small ribosomal subunit joining, aminoacyl tRNA transportation, and translation control (Pachler et al., 2004; Sulima et al., 2014). In human, RPL10 is also known as the QM protein, which could be translocated from the cytoplasm to the nucleus promoted by presenilin 1 (PS1). The nuclear QM could bind to c-Jun protein as a negative regulator of the latter (Imafuku et al., 1999; Pollutri and Penzo, 2020). There are three paralogous genes encoding RPL10 in the *Arabidopsis* genome [*RPL10A* (*At1G14320*), *RPL10B* (*AT1G26910*), and *RPL10C* (*AT1G66580*)], with these genes sharing 90–95% sequence identity (Barakat et al., 2001). The concept of RPL10A functioning apart from the ribosome was first demonstrated with the finding that it is a substrate for NIK1 (NSP-interacting kinase, a leucine-rich repeat receptor-like kinase) (Carvalho et al., 2008). RPL10A could be phosphorylated by NIK1, then phosphorylated RPL10A could be transported from the cytoplasm into the nucleus where it may function as a regulator to activate the viral defense pathway. Loss of function of RPL10A results in the same susceptibility to geminivirus infections as that observed in *nik1* null mutants (Carvalho et al., 2008). The exact mechanism of NIK1-mediated antiviral signaling was unclear until 2015, at which point Cristiane Zorzatto et al. observed that as a response to viral infection the NIK1 kinase was constitutively activated. The active NIK1 kinase phosphorylates its substrate RPL10A in the cytoplasm. The phosphorylation of RPL10A promotes its nucleus localization. After that it interacts with a putative transcription factor, L10-interacting MYB domain-containing protein (LIMYB), which may lower the transcriptional speed of genes related to the translational-machinery such as RP genes. Down-regulation of the transcription of the translational-machinery genes could cause global translation suppression, which could be used as an antiviral immunity strategy (Zorzatto et al., 2015). It is unclear as to whether RPL10B or RPL10C have similar functions of anti-virus defense.

In addition to the anti-virus defense mechanisms, *Arabidopsis RPL10* genes have also been reported to respond to UV-B (ultraviolet-B) stress differently: RPL10A has no response, RPL10B is down-regulated, and RPL10C is up-regulated under UV-B treatments (Ferreyra et al., 2010b). The different UV-B responses of the paralogous *RPL10* genes suggest that functional specializations exist among them. However, further studies should be performed to demonstrate whether this is a novel extraribosomal function of RPL10.

### Free RPL24B Participates in miRNA Biogenesis

**FIGURE 1:**
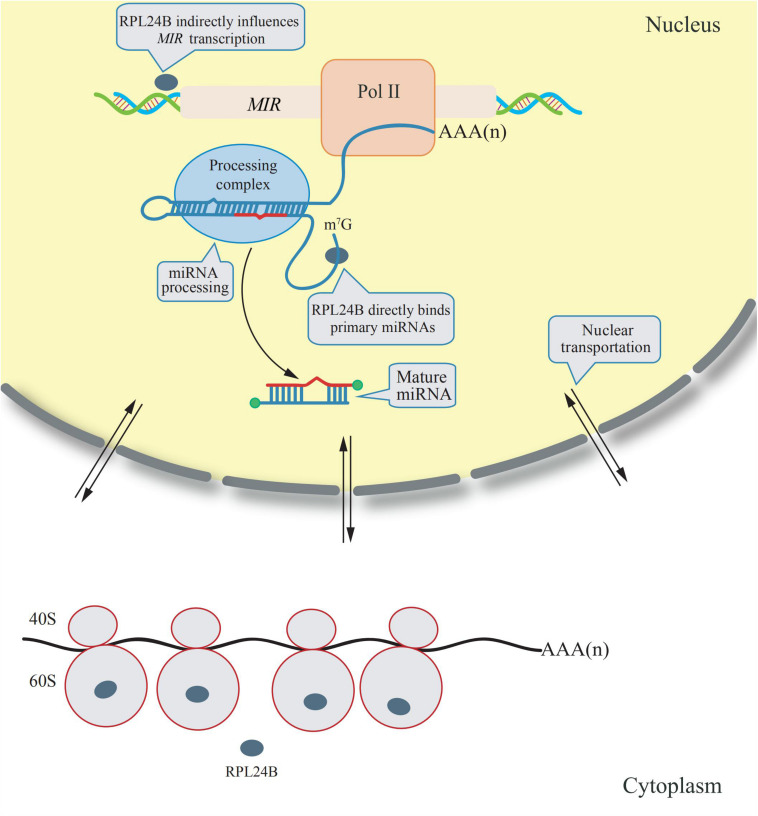
Extraribosomal functions of RPL24B in *Arabidopsis*. RPL24B was translated by cytosolic ribsomes and a portion of RPL24B was translocated from the cytoplasm into the nucleus. In nucleus, RPL24B indirectly influences *MIR* transcription and directly binds primary miRNAs promoting their processing (Li et al., 2017).

The RPL24 is a component of ribosomes from archaebacterium and eukaryote, but not from eubacteria. It is located on the surface of the ribosomal large subunit that mediate the interaction between the large and small ribosomal subunits, as studied on the crystal structure of ribsomes in archaebacteria (Ban et al., 2000). *Arabidopsis* has two RPL24 homologs: *RPL24A* (*AT2G36620*) and *RPL24B* (*AT3G53020*) (Barakat et al., 2001). RPL24B, also known as SHORT VALVE 1 (STV1), is essential for the translation initiation of several proteins, including ETTIN (ETT) and MONOPTEROS (MP) (two auxin response factors), and it has been reported to be involved in gynoecium patterning (Nishimura et al., 2005). However, a novel function of RPL24B was discovered in 2017; it was observed that RPL24B functions in miRNA biogenesis in *Arabidopsis* (Li et al., 2017). RPL24B is originally translated in the cytoplasm by ribosomes, a fraction of RPL24B proteins are imported into the nucleus, in which primary miRNAs are transcribed and processed into mature miRNAs. In *rpl24b* mutants, the accumulation of primary miRNA transcripts is reduced, however, RPL24B neither interacts with the Pol II RNA polymerase nor binds to the promoter region of miRNAs, suggesting RPL24B plays an indirect role to promote *MIR* genes transcription. In addition, RPL24B were demonstrated to bind directly to the primary miRNA transcripts but does not associate with the miRNA processing complex, indicating that it could facilitate the recruitment of primary miRNA transcripts to the processing complex (Li et al., 2017). A diagram explaining the mechanism of these extraribosomal functions of RPL24B is showing in Figure 1.

### RPS6 Plays Roles in rDNA Transcription

RPS6 has been demonstrated to be a key downstream effector of the TOR (target of rapamycin) signaling pathway, which is conserved among yeast, mammals, insects, and plants (Hay and Sonenberg, 2004; Mahfouz et al., 2006; Chen et al., 2018). The TOR-dependent phosphorylation of RPS6 within ribosomes may lead to enhanced translation (Chen et al., 2018). There are two paralogous genes [*RPS6A* (*At4g31700*) and *RPS6B* (*At5g10360*)] encoding RPS6 in *Arabidopsis*, these two genes are demonstrated to have the same function and one gene could complement the loss of function phenotype of another gene (Creff et al., 2010). Free RPS6 was proved to be a component of a multiprotein complex which contains many protein subunits involved in the chromatin-related activities (Kim et al., 2014). Interestingly, AtHD2 (histone deacetylase 2), which had been implicated in silencing of rDNA transcription in *Arabidopsis*, was also presented in this complex. When expressed alone in protoplasts, both GFP-tagged AtHD2 and RPS6 were localized in the nucleus. In contrast, co-expression of RPS6 with AtHD2 caused a change in their localization from the nucleus to the nucleolus. The interaction between AtHD2 and RPS6 was proved to play a negative role in the regulation of rDNA transcription. In addition, free RPS6 directly binds to the rRNA gene promoter in *Arabidopsis* (as suggested by a ChIP analysis), which indicates another extraribosomal function of RPS6 (Kim et al., 2014).

### Plant-Specific RPP3 Acts as a Chaperone for Both Protein and RNA

RPP proteins (ribosomal protein P) form a lateral protuberance on the large ribosomal subunit (Szick et al., 1998). Plant RPP proteins include the RPP1, RPP2, and RPP3 families, the last of which is specific to plants. The *Arabidopsis* genome encodes two paralogous *RPP3* genes, designated as *RPP3A* (*At4g25890*) and *RPP3B* (*At5g57290*). Most of the investigated properties of *RPP3A* and *RPP3B* are indistinguishable with each other (Kang et al., 2016). Recently, RPP3 was suggested to be a novel protein component of high-molecular weight (HMW) complexes, which are formed from HSPs (heat shock proteins). RPP3 was also demonstrated to exhibit both protein and RNA chaperone activities to increase the tolerance of plants to heat and cold stresses (Kang et al., 2016).

### Free RACK1 Acts in Plant Immunity

RACK1 (receptor of activated C kinase) has previously been implicated as a scaffold involved in many signal transduction functions (Kadrmas et al., 2007). Due to the improved methods of mass spectrometry, RACK1 has been added to the eukaryotic ribosome as a newly discovered factor (Link et al., 1999; Carroll, 2013). There are three copies of the *RACK1* gene [*RACK1A* (*At1g18080*), *RACK1B* (*At1g48630*), and *RACK1C* (*At3g18130*)] in *Arabidopsis* (Guo and Chen, 2008). In principle, RACK1B and RACK1C are functionally equivalent to RACK1A but the expression level of *RACK1A* is much higher than the levels of either *RACK1B* or *RACK1C* (Guo and Chen, 2008). As a component of the cytosolic ribosome, RACK1 has been demonstrated to serve a key role in 80S ribosome assembly. It is phosphorylated by the activated C kinase and directs the release of eIF6 (eukaryotic translation initiation factor 6) from the 60S subunit, thus allowing for the assembly of the 80S ribosome (Ceci et al., 2003). RACK1 has also been demonstrated to be a key integrator and mediator of hormonal control of translation in many studies (Chen et al., 2006; Guo and Chen, 2008; Guo et al., 2009, 2011). Furthermore, being a scaffold protein, free RACK1 was identified as the first plant MAPK (mitogen-activated protein kinase) scaffold protein that connects heterotrimeric G protein with a MAPK cascade to form a unique signaling pathway in plant immunity (Cheng et al., 2015), which represents an extraribosomal function of RACK1.

## Possible Extraribosomal Functions of RPs Need Further Investigations

In fact, a collection of protein-protein interaction studies (MS spectrometry after immunoprecipitation (IP), yeast two-hybrid system) and genetic screens (rescue, suppression or enhancement of a specific phenotype when introduce another mutation) have identified many other RPs (or their genes) that function in different types of physiological activities. However, the mechanism underlying their physiological activities and whether these functions are performed by extraribosomal functions remain unclear.

### Involvement of RPS27 in mRNA Degradation

As mentioned above, RPS27 has a zinc finger motif which may confer it with abilities to interact with non-ribosomal components such as DNA or mRNA. RPS27, also known as Metallopanstimulin-1 (MPS1) in human, has been used as a liquid biopsy marker since it could be secreted into extracellular fluids of patients with different cancers (Fernandez-Pol, 2016). Abnormal high expression level of RPS27 is observed in several malignant tumors (Fernandez-Pol, 2012). *RPS27B* (*AT3G61110*), which is one of the three RPS27 paralogs in *Arabidopsis*, has been suggested to act as a regulator of transcript stability in response to genotoxic treatments (Revenkova et al., 1999). The proposed mechanism is that RPS27B is involved in the degradation of damaged RNAs (induced by genotoxic treatments) (Revenkova et al., 1999).

### RPL5 Serves as a Novel Positive Trans-Regulator of the Telomere Length Set Point

The nuclear re-entry of 5S rRNA in the ribosome has been observed to be exclusively mediated by the RPL5 protein (Rudt and Pieler, 1996). *RPL5A* (*At3g25520*) and *RPL5B* (*At5g39740*) are two paralogous genes encoding RPL5 in the *Arabidopsis* genome (Barakat et al., 2001). The functions of these two paralogous genes may be identical because the phenotypes of *rpl5a* and *rpl5b* are similar (Fujikura et al., 2009). Interestingly, RPL5A and RPL5B have been reported to interact with NOP2A (a ribosomal RNA methyltransferase with major roles in cellular proliferation); all of these three factors play a role in the positive regulation of the telomere length set point (Abdulkina et al., 2019), which may represent an extraribosomal function of RPL5.

### RPL22 Regulates Symbiotic Nodulation in *Robinia pseudoacacia*

*Rpf84*, which was identified from the leguminous tree *Robinia pseudoacacia*, is a homolog of *RPL22*. RPL22 is an external, salt-extractable component on the surface of the 60S ribosomal large subunit and is positioned away from the subunit interface (Fahl et al., 2015). In addition, RPL22 has been demonstrated to function as a tumor suppressor in animals, which may represent an extraribosomal function (Fahl et al., 2015). RPL22 was found to be localized not only in the cytoplasm, but also in the nucleus. Knockdown the expression of RPL22 by RNAi results in severe damage of nodule in *Robinia pseudoacacia.* In contrast, overexpression of RPL22 could promote the nodule formation and delay the aging process of nodules (Feng et al., 2019).

### RPL30 Modulates Cell Division and Size in *Chlamydomonas*

Using immunoprecipitation (IP) in combination with MS spectrometry, RPL30 in *Chlamydomonas* was isolated and identified as a SMT7 (suppressor of *mat3* 7) interacting protein whose SUMO deconjugation is regulated by SMT7 (Lin et al., 2020). As a result of this interaction, a defect in SMT7 caused increased levels of RPL30 SUMOylation, reducing the number of cell divisions and increasing cell size. It is unclear how increased dose of SUMO-conjugated RPL30 protein affects cell division. One possibility is that the SUMOylation status of RPL30 protein modulates RPL30-related ribosome biogenesis or preribosomal particle assembly, translocation, or maturation. Another possibility is that accumulation of SUMOylated RPL30 has an extraribosomal function.

### RPS27a and RPL40 Are Ubiquitin-Extension Protein*s*

Intriguingly, the 76-aa ubiquitin protein is fused (in-frame) to two different ribosomal proteins (RPS27a and RPL40) in plants but is immediately cleaved after translation (Callis, 2014), and this phenomenon is highly conserved between different organisms. The products of these RP genes are important sources of ubiquitin. In yeast, there are evidences suggesting that covalent association of these RPs to ubiquitin could promote the formation of special cellular structure to facilitate pre-rRNA processing and ribosome assembly (Finley et al., 1989). In *Arabidopsis*, *RPL40A* (*At2g36170*), *RPL40B* (*At3g52590*), *RPS27aA* (*At1g23410*), *RPS27aB* (*At2g47110*), and *RPS27aC* (*At3g62250*) are designated as *UBQ1*, *UBQ2*, *UBQ17*, *UBQ5*, and *UBQ6*, respectively.

### RPs Involved in Plant Development and Stress Responses

*Arabidopsis* RPS15aE has been demonstrated to have a potential role as a growth regulator (Szick-Miranda et al., 2010). It was reported that RPL18aB was involved in sexual reproduction and played a critical role in male gametophyte development and embryo pattern formation (Yan et al., 2016). RPL14B has been found to be critical for fertilization in *Arabidopsis* (Luo et al., 2020). In addition to its previously mentioned extraribosomal function, RPL5 from *Arabidopsis* was also observed to bind the *potato spindle tuber viroid* (PSTVd) RNA *in vitro* (Eiras et al., 2011).

**FIGURE 2:**
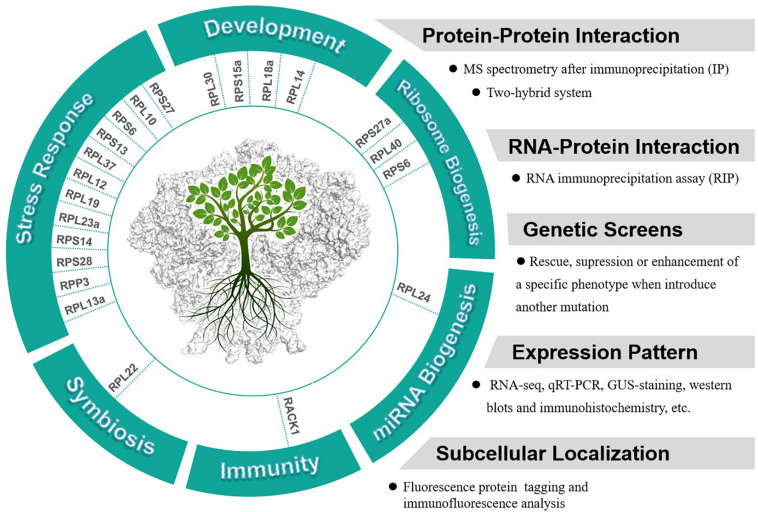
Potential extraribosomal functions of cytosolic RPs in plants and relevant methods and appraoches to analyze extraribosomal function of RPs.

It is notable that many RPs have been reported to respond to abiotic or biotic stresses in different plants. For examples, three ribosomal protein genes, *RPS13*, *RPS6*, and *RPL37* were induced by a low temperature treatment in soybeans (Kim et al., 2004). Overexpression of an eggplant RPL13a in potato has been observed to enhance the resistance to *Verticillium dahliae* (Yang et al., 2013). Both RPL12 and RPL19 have been reported to serve a role in non-host disease resistance against bacterial pathogens in *N. benthamiana* (Nagaraj et al., 2016). Overexpression of RPL23a in transgenic rice plants can increase water-use efficiency and tolerance to drought and salt stresses (Moin et al., 2017). RPS14 was reported to be up-regulated by cytokinins and down-regulated by abscisic acid in detached lupin cotyledons (Cherepneva et al., 1998). Expression of RPS28 was found to be down-regulated in both seedling roots and shoots in response to drought, high salinity, or abscisic acid in *Asteraceae* (Liu and Baird, 2003). In summary, studies indicate that many plant RPs are involved in plant development and stress responses, further investigations are needed to confirm whether the functions of these RPs in these processes are extraribosomal. We collected current knowledge about the extraribosomal functions of RPs (with both substantial and circumstantial evidences) in plants and suggested the approaches to study these functions (Figure 2).

## Future Challenges

### Extensive Duplicated RP Genes in Plants May Provide Rich Source of Extraribosomal Functions

The organization of the RP genes is diversified in different organisms. In *Escherichia coli* and mammals, most of the ribosomal protein families contain only one functional ribosomal protein (Lindahl and Zengel, 1986; Uechi et al., 2001). By contrast, in yeast, 3/4 RP families are encoded by two functional members, which arose from the ancient whole-genome duplication event in the yeast lineage (Wolfe and Shields, 1997). The plant RP families are apparently more complex, this is probably caused by several round of ancient whole-genome duplication (paleopolyploidy) and species-specific whole-genome duplication (polyploidy) events in the evolutionary history of plants, which is recognized as an important characteristic of plant genome evolution (Alix et al., 2017; Van de Peer et al., 2017). For example, there are probably 249 genes of the 81 RP families in the genome of *Arabidopsis* (Barakat et al., 2001; Fokkens et al., 2012). While at least 996 RP genes are identified in *Brassica napus*, which experienced an extra genome triplication event in the Brassica lineages after divergence from *Arabidopsis* (Lysak et al., 2005; Whittle and Krochko, 2009). The tremendous increase in members of each RP family and complexity of the duplication mechanisms suggested that the roles of plant RP genes may be far more complex than we expected as components of protein-making machines.

Indeed, increasing evidences have indicated that, on the basis of maintaining their ribosomal functions, one of the paralogous RPs may be allowed the freedom for evolving new functions. For example, it has been shown that deletions of certain copy of the yeast paralogs caused divergent phenotypes in various cell development and responses to virus and drugs, suggesting that these yeast paralogs may have divergent functions (Ohtake and Wickner, 1995; Ni and Snyder, 2001; Enyenihi and Saunders, 2003; Zettel et al., 2003). In plants, marked divergences in expression patterns and subcellular localization patterns have been found within the duplicate gene pairs. For example, the expression analysis revealed 45 of 55 RP families in *Arabidopsis* comprised various degrees of expression divergences, and differential expression patterns have been detected in more than two-thirds of the RP families in *Brassica napus* (Whittle and Krochko, 2009; Savada and Bonhamsmith, 2014). The three members of *Arabidopsis* RPL10 were found to exhibit totally different responses and differential subcellular localizations under UV-B (ultraviolet-B) treatment, indicating selectively requirement of certain RPL10 member for UV-B stress response (Ferreyra et al., 2010a; Zhang et al., 2020). This phenomenon of RPL10 was also observed in maize (Zea mays), suggesting that it is a universal function in plants (Ferreyra et al., 2010b). Although unique functions for individual RP paralogs in plants are still poorly understood, growing evidences have shown us the tip of the iceberg.

### More Viable Mutant of RP Gene Alleles Will Be Available

Genome-wide genetic screen searching for genes involved in embryo development had identified a number of RP gene mutants as *EMB* (EMBRYO DEFECTIVE) genes: *RPL3A*, *RPL8A*, *RPL19A*, *RPL23C*, *RPL40B*, and *RPS11A* were assigned as *EMB2207*, *EMB2296*, *EMB2386*, *EMB2171*, *EMB2167*, and *EMB1080*, respectively (Meinke, 2020). Lacking viable mutant of these RP genes hinders researches on how exactly these RP act inside and outside the ribosome. It is generally thought that mutations in these RP genes result in the amount of functional ribosome decreasing below a threshold which could support embryo development. So far, only approximately 20 RP mutants have been well characterized in plants (Browning and Bailey-Serres, 2015). However, the rapid development of gene editing technology, such as CRISPR/CAS9, will lead to increased availability of hypomorphic mutant alleles of more RP genes, for complete loss of function of which will cause embryo lethality.

In summary, although it is not easy to detect the extraribosomal function of RPs, several RPs have indeed been shown to play other molecular and biochemical roles in addition to their contributions to translation. We believe that the extraribosomal functions of plant RPs discovered so far represent only the tip of the iceberg. We envision that more extraribosomal functions of plant RPs will be identified in the future.

## Author Contributions

BM planned this work. WX and TL wrote the manuscript. All authors contributed to the article and approved the submitted version.

## Conflict of Interest

The authors declare that the research was conducted in the absence of any commercial or financial relationships that could be construed as a potential conflict of interest.

## References

[B1] Abdulkina L. R. Kobayashi C. Lovell J. T. Chastukhina I. B. Aklilu B. B. Agabekian I. A. (2019). Components of the ribosome biogenesis pathway underlie establishment of telomere length set point in *Arabidopsis*. *Nat. Commun.* 10:5479. 10.1038/s41467-019-13448-z PMC6889149 31792215

[B2] Alix K. Gerard P. R. Schwarzacher T. Heslop-Harrison J. S. P. (2017). Polyploidy and interspecific hybridization: partners for adaptation, speciation and evolution in plants. *Ann. Bot.* 120 183–194. 10.1093/aob/mcx079 28854567 PMC5737848

[B3] Ban N. Nissen P. Hansen J. Moore P. B. Steitz T. A. (2000). The complete atomic structure of the large ribosomal subunit at 2.4 Å resolution. *Science* 289 905–920. 10.1126/science.289.5481.905 10937989

[B4] Barakat A. Szickmiranda K. Chang I. Guyot R. Blanc G. Cooke R. (2001). The organization of cytoplasmic ribosomal protein genes in the *Arabidopsis* genome. *Plant Physiol.* 127 398–415. 10.1104/pp.127.2.398 11598216 PMC125077

[B5] Blumenthal T. Carmichael G. G. (1979). RNA replication: function and structure of Qbeta-replicase. *Annu. Rev. Biochem.* 48 525–548. 10.1146/annurev.bi.48.070179.002521 382992

[B6] Bohnsack K. E. Bohnsack M. T. (2019). Uncovering the assembly pathway of human ribosomes and its emerging links to disease. *EMBO J.* 38:e100278. 10.15252/embj.2018100278 PMC6600647 31268599

[B7] Brodersen D. E. Nissen P. (2005). The social life of ribosomal proteins. *FEBS J.* 272 2098–2108. 10.1111/j.1742-4658.2005.04651.x 15853795

[B8] Browning K. S. Bailey-Serres J. (2015). Mechanism of cytoplasmic mRNA translation. *Arabidopsis Book* 13:e0176. 10.1199/tab.0176 26019692 PMC4441251

[B9] Callis J. (2014). The ubiquitination machinery of the ubiquitin system. *Arabidopsis Book* 12:e0174. 10.1199/tab.0174 25320573 PMC4196676

[B10] Carroll A. J. (2013). The *Arabidopsis* cytosolic ribosomal proteome: from form to function. *Front. Plant Sci.* 4:32. 10.3389/fpls.2013.00032 23459595 PMC3585428

[B11] Carvalho C. M. Santos A. A. Pires S. R. Rocha C. S. Saraiva D. I. Machado J. P. B. (2008). Regulated nuclear trafficking of rpL10A mediated by NIK1 represents a defense strategy of plant cells against virus. *PLoS Pathog.* 4:e1000247. 10.1371/journal.ppat.1000247 19112492 PMC2597721

[B12] Ceci M. Gaviraghi C. Gorrini C. Sala L. A. Offenhäuser N. Carlo Marchisio P. (2003). Release of eIF6 (p27BBP) from the 60S subunit allows 80S ribosome assembly. *Nature* 426 579–584. 10.1038/nature02160 14654845

[B13] Chan Y. L. Suzuki K. Olvera J. Wool I. G. (1993). Zinc finger-like motifs in rat ribosomal-proteins S27 and S29. *Nucleic Acids Res.* 21 649–655. 10.1093/nar/21.3.649 8441676 PMC309165

[B14] Chang I. Szickmiranda K. Pan S. Baileyserres J. (2005). Proteomic characterization of evolutionarily conserved and variable proteins of *Arabidopsis* cytosolic ribosomes. *Plant Physiol.* 137 848–862. 10.1104/pp.104.053637 15734919 PMC1065386

[B15] Chen G. Liu M. Xiong Y. Sheen J. Wu S. (2018). TOR and RPS6 transmit light signals to enhance protein translation in deetiolating *Arabidopsis* seedlings. *Proc. Natl. Acad. Sci. U.S.A.* 115 12823–12828. 10.1073/pnas.1809526115 30482859 PMC6294885

[B16] Chen J. Ullah H. Temple B. Liang J. Guo J. Alonso J. M. (2006). RACK1 mediates multiple hormone responsiveness and developmental processes in *Arabidopsis*. *J. Exp. Bot.* 57 2697–2708. 10.1093/jxb/erl035 16829549

[B17] Cheng Z. Li J. Niu Y. Zhang X. Woody O. Z. Xiong Y. (2015). Pathogen-secreted proteases activate a novel plant immune pathway. *Nature* 521 213–216. 10.1038/nature14243 25731164 PMC4433409

[B18] Cherepneva G. N. Oelmüller R. Kulaeva O. N. Kusnetsov V. V. (1998). Expression of the ribosomal protein S14 in lupin cotyledons is stimulated by cytokinin and inhibited by abscisic acid and light. *Bot. Acta* 111 287–290. 10.1111/j.1438-8677.1998.tb00710.x

[B19] Creff A. Sormani R. Desnos T. (2010). The two *Arabidopsis* RPS6genes, encoding for cytoplasmic ribosomal proteins S6, are functionally equivalent. *Plant Mol. Biol.* 73 533–546. 10.1007/s11103-010-9639-y 20437080

[B20] Eiras M. Nohales M. A. Kitajima E. W. Flores R. Daros J. (2011). Ribosomal protein L5 and transcription factor IIIA from *Arabidopsis thaliana* bind in vitro specifically Potato spindle tuber viroid RNA. *Arch. Virol.* 156 529–533. 10.1007/s00705-010-0867-x 21153748

[B21] Enyenihi A. H. Saunders W. S. (2003). Large-scale functional genomic analysis of sporulation and meiosis in *Saccharomyces cerevisiae*. *Genetics* 163 47–54. 12586695 10.1093/genetics/163.1.47 PMC1462418

[B22] Fahl S. P. Wang M. Zhang Y. Duc A. C. Wiest D. L. (2015). Regulatory roles of Rpl22 in hematopoiesis: an old dog with new tricks. *Crit. Rev. Immunol.* 35 379–400. 10.1615/critrevimmunol.v35.i5.30 26853850 PMC5111805

[B23] Feng Z. Zhang L. Wu Y. Wang L. Xu M. Yang M. (2019). The Rpf84 gene, encoding a ribosomal large subunit protein, RPL22, regulates symbiotic nodulation in *Robinia pseudoacacia*. *Planta* 250 1897–1910. 10.1007/s00425-019-03267-3 31485773

[B24] Fernandez-Pol J. A. (2012). Increased serum level of RPMPS-1/S27 protein in patients with various types of cancer is useful for the early detection, prevention and therapy. *Cancer Genomics Proteomics* 9 203–256. 22798506

[B25] Fernandez-Pol J. A. (2016). A novel marker for purkinje cells, ribosomal protein MPS1/S27: expression of MPS1 in human cerebellum. *Cancer Genomics Proteomics* 13 47–53. 26708598

[B26] Ferreiracerca S. Poll G. Kuhn H. Neueder A. Jakob S. Tschochner H. (2007). Analysis of the in vivo assembly pathway of eukaryotic 40s ribosomal proteins. *Mol. Cell* 28 446–457. 10.1016/j.molcel.2007.09.029 17996708

[B27] Ferreyra M. L. F. Biarc J. Burlingame A. L. Casati P. (2010a). *Arabidopsis* L10 ribosomal proteins in UV-B responses. *Plant Signal. Behav.* 5 1222–1225. 10.4161/psb.5.10.12758 20855946 PMC3115351

[B28] Ferreyra M. L. F. Pezza A. Biarc J. Burlingame A. L. Casati P. (2010b). Plant L10 ribosomal proteins have different roles during development and translation under ultraviolet-B stress. *Plant Physiol.* 153 1878–1894. 10.1104/pp.110.157057 20516338 PMC2923885

[B29] Finley D. Bartel B. Varshavsky A. (1989). The tails of ubiquitin precursors are ribosomal proteins whose fusion to ubiquitin facilitates ribosome biogenesis. *Nature* 338 394–401. 10.1038/338394a0 2538753

[B30] Fokkens L. Hogeweg P. Snel B. (2012). Gene duplications contribute to the overrepresentation of interactions between proteins of a similar age. *BMC Evol. Biol.* 12:99. 10.1186/1471-2148-12-99 22732003 PMC3457867

[B31] Fujikura U. Horiguchi G. Ponce M. R. Micol J. L. Tsukaya H. (2009). Coordination of cell proliferation and cell expansion mediated by ribosome-related processes in the leaves of *Arabidopsis thaliana*. *Plant J.* 59 499–508. 10.1111/j.1365-313x.2009.03886.x 19392710

[B32] Giavalisco P. Wilson D. Kreitler T. Lehrach H. Klose J. Gobom J. (2005). High heterogeneity within the ribosomal proteins of the *Arabidopsis thaliana* 80S ribosome. *Plant Mol. Biol.* 57 577–591. 10.1007/s11103-005-0699-3 15821981

[B33] Green R. Noller H. F. (1997). Ribosomes and translation. *Annu. Rev. Biochem.* 66 679–716. 9242921 10.1146/annurev.biochem.66.1.679

[B34] Guo J. Chen J. (2008). RACK1 genes regulate plant development with unequal genetic redundancy in *Arabidopsis*. *BMC Plant Biol.* 8:108. 10.1186/1471-2229-8-108 18947417 PMC2577656

[B35] Guo J. Wang J. Xi L. Huang W. Liang J. Chen J. (2009). RACK1 is a negative regulator of ABA responses in *Arabidopsis*. *J. Exp. Bot.* 60 3819–3833. 10.1093/jxb/erp221 19584117 PMC2736894

[B36] Guo J. Wang S. Valerius O. Hall H. Zeng Q. Li J. (2011). Involvement of *Arabidopsis* RACK1 in protein translation and its regulation by abscisic acid. *Plant Physiol.* 155 370–383. 10.1104/pp.110.160663 21098678 PMC3075769

[B37] Hay N. Sonenberg N. (2004). Upstream and downstream of mTOR. *Genes Dev.* 18 1926–1945. 15314020 10.1101/gad.1212704

[B38] Horiguchi G. Mollamorales A. Perezperez J. M. Kojima K. Robles P. Ponce M. R. (2011). Differential contributions of ribosomal protein genes to *Arabidopsis thaliana* leaf development. *Plant J.* 65 724–736. 10.1111/j.1365-313x.2010.04457.x 21251100

[B39] Imafuku I. Masaki T. Waragai M. Takeuchi S. Kawabata M. Hirai S.-I. (1999). Presenilin 1 suppresses the function of C-Jun homodimers via interaction with Qm/Jif-1. *J. Cell Biol.* 147 121–134. 10.1083/jcb.147.1.121 10508860 PMC2164975

[B40] Isalan M. (2020). *Zinc Fingers: Structure and Design✩. Reference Module in Life Sciences.* Amsterdam: Elsevier.

[B41] Kadrmas J. L. Smith M. A. Pronovost S. M. Beckerle M. C. (2007). Characterization of RACK1 function in *Drosophila* development. *Dev. Dyn.* 236 2207–2215. 10.1002/dvdy.21217 17584887

[B42] Kang C. H. Lee Y. M. Park J. H. Nawkar G. M. Oh H. T. Kim M. G. (2016). Ribosomal P3 protein AtP3B of *Arabidopsis* acts as both protein and RNA chaperone to increase tolerance of heat and cold stresses. *Plant Cell Environ.* 39 1631–1642. 10.1111/pce.12742 27004478

[B43] Kim K. Park S. Chung Y. Chung C. Kim J. Lee J. (2004). Molecular cloning of low-temperature-inducible ribosomal proteins from soybean. *J. Exp. Bot.* 55 1153–1155. 10.1093/jxb/erh125 15020631

[B44] Kim Y. Kim S. Shin Y. Hur Y. Kim W. Lee M. (2014). Ribosomal protein S6, a target of rapamycin, is involved in the regulation of rRNA genes by possible epigenetic changes in *Arabidopsis*. *J. Biol. Chem.* 289 3901–3912. 10.1074/jbc.m113.515015 24302738 PMC3924259

[B45] Klein D. Moore P. B. Steitz T. A. (2004). The roles of ribosomal proteins in the structure, assembly and evolution of the large ribosomal subunit. *J. Mol. Biol.* 340 141–177. 10.1016/j.jmb.2004.03.076 15184028

[B46] Kuhn J. F. Tran E. J. Maxwell E. S. (2002). Archaeal ribosomal protein L7 is a functional homolog of the eukaryotic 15.5kD/Snu13p snoRNP core protein. *Nucleic Acids Res.* 30 931–941. 10.1093/nar/30.4.931 11842104 PMC100351

[B47] Li S. Liu K. Zhang S. Wang X. Yu B. (2017). STV1, a ribosomal protein, binds primary microRNA transcripts to promote their interaction with the processing complex in *Arabidopsis*. *Proc. Natl. Acad. Sci. U.S.A.* 114:201613069. 10.1073/pnas.1613069114 PMC5307444 28115696

[B48] Lin Y.-L. Chung C.-L. Chen M.-H. Chen C.-H. Fang S.-C. (2020). SUMO protease SMT7 modulates ribosomal protein L30 and regulates cell-size checkpoint function. *Plant Cell* 32 1285–1307. 10.1105/tpc.19.00301 32060174 PMC7145494

[B49] Lindahl L. Zengel J. M. (1986). Ribosomal genes in *Escherichia coli*. *Annu. Rev. Genet.* 20 297–326. 10.1146/annurev.ge.20.120186.001501 2434021

[B50] Lindstrom M. S. (2009). Emerging functions of ribosomal proteins in gene-specific transcription and translation. *Biochem. Biophys. Res. Commun.* 379 167–170. 10.1016/j.bbrc.2008.12.083 19114035

[B51] Link A. J. Eng J. K. Schieltz D. Carmack E. Mize G. J. Morris D. R. (1999). Direct analysis of protein complexes using mass spectrometry. *Nat. Biotechnol.* 17 676–682. 10404161 10.1038/10890

[B52] Liu X. Baird W. V. (2003). The ribosomal small-subunit protein S28 gene from *Helianthus annuus* (Asteraceae) is down-regulated in response to drought, high salinity, and abscisic acid. *Am. J. Bot.* 90 526–531. 10.3732/ajb.90.4.526 21659145

[B53] Luo A. Zhan H. Zhang X. Du H. Zhang Y. Peng X. (2020). Cytoplasmic ribosomal protein L14B is essential for fertilization in *Arabidopsis*. *Plant Sci.* 292:110394. 10.1016/j.plantsci.2019.110394 32005399

[B54] Lynch M. Conery J. S. (2000). The evolutionary fate and consequences of duplicate genes. *Science* 290 1151–1155. 10.1126/science.290.5494.1151 11073452

[B55] Lysak M. A. Koch M. A. Pecinka A. Schubert I. (2005). Chromosome triplication found across the tribe Brassiceae. *Genome Res.* 15 516–525. 10.1101/gr.3531105 15781573 PMC1074366

[B56] Mahfouz M. M. Kim S. Delauney A. J. Verma D. P. S. (2006). *Arabidopsis* target of rapamycin interacts with RAPTOR, which regulates the activity of S6 kinase in response to osmotic stress signals. *Plant Cell* 18 477–490. 10.1105/tpc.105.035931 16377759 PMC1356553

[B57] Meinke D. W. (2020). Genome-wide identification of EMBRYO-DEFECTIVE (EMB) genes required for growth and development in *Arabidopsis*. *New Phytol.* 226 306–325. 10.1111/nph.16071 31334862

[B58] Moin M. Bakshi A. Madhav M. S. Kirti P. B. (2017). Expression profiling of ribosomal protein gene family in dehydration stress responses and characterization of transgenic rice plants overexpressing RPL23A for water-use efficiency and tolerance to drought and salt stresses. *Front. Chem.* 5:97. 10.3389/fchem.2017.00097 29184886 PMC5694489

[B59] Molavi G. Samadi N. Hosseingholi E. Z. (2019). The roles of moonlight ribosomal proteins in the development of human cancers. *J. Cell. Physiol.* 234 8327–8341. 10.1002/jcp.27722 30417503

[B60] Moore P. B. Steitz T. A. (2011). The roles of RNA in the synthesis of protein. *Cold Spring Harb. Perspect. Biol.* 3:a003780. 10.1101/cshperspect.a003780 PMC3220363 21068149

[B61] Nagaraj S. Senthilkumar M. Ramu V. S. Wang K. Mysore K. S. (2016). Plant ribosomal proteins, RPL12 and RPL19, play a role in nonhost disease resistance against bacterial pathogens. *Front. Plant Sci.* 6:1192. 10.3389/fpls.2015.01192 26779226 PMC4702080

[B62] Naora H. Naora H. (1999). Involvement of ribosomal proteins in regulating cell growth and apoptosis: translational modulation or recruitment for extraribosomal activity? *Immunol. Cell Biol.* 77 197–205. 10.1046/j.1440-1711.1999.00816.x 10361251

[B63] Natchiar S. K. Myasnikov A. G. Kratzat H. Hazemann I. Klaholz B. P. (2017). Visualization of chemical modifications in the human 80S ribosome structure. *Nature* 551 472–477. 10.1038/nature24482 29143818

[B64] Ni L. Snyder M. (2001). A genomic study of the bipolar bud site selection pattern in *Saccharomyces cerevisiae*. *Mol. Biol. Cell* 12 2147–2170. 10.1091/mbc.12.7.2147 11452010 PMC55669

[B65] Nishimura T. Wada T. Yamamoto K. T. Okada K. (2005). The *Arabidopsis* STV1 protein, responsible for translation reinitiation, is required for auxin-mediated gynoecium patterning. *Plant Cell* 17 2940–2953. 10.1105/tpc.105.036533 16227452 PMC1276021

[B66] Ohtake Y. Wickner R. B. (1995). Yeast virus propagation depends critically on free 60S ribosomal subunit concentration. *Mol. Cell. Biol.* 15 2772–2781. 10.1128/mcb.15.5.2772 7739558 PMC230508

[B67] Pachler K. Karl T. Kolmann K. Mehlmer N. Eder M. Loeffler M. (2004). Functional interaction in establishment of ribosomal integrity between small subunit protein rpS6 and translational regulator rpL10/Grc5p. *FEMS Yeast Res.* 5 271–280. 10.1016/j.femsyr.2004.07.009 15556089

[B68] Pollutri D. Penzo M. (2020). Ribosomal protein L10: from function to dysfunction. *Cells* 9:2503. 10.3390/cells9112503 33227977 PMC7699173

[B69] Revenkova E. Masson J. Koncz C. Afsar K. Jakovleva L. Paszkowski J. (1999). Involvement of *Arabidopsis thaliana* ribosomal protein S27 in mRNA degradation triggered by genotoxic stress. *EMBO J.* 18 490–499. 10.1093/emboj/18.2.490 9889204 PMC1171142

[B70] Rosado A. Li R. van de Ven W. Hsu E. Raikhel N. V. (2012). *Arabidopsis* ribosomal proteins control developmental programs through translational regulation of auxin response factors. *Proc. Natl. Acad. Sci. U.S.A.* 109 19537–19544. 10.1073/pnas.1214774109 23144218 PMC3511758

[B71] Ross C. L. N. Patel R. R. Mendelson T. C. Ware V. C. (2007). Functional conservation between structurally diverse ribosomal proteins from *Drosophila melanogaster* and *Saccharomyces cerevisiae*: fly L23a can substitute for yeast L25 in ribosome assembly and function. *Nucleic Acids Res.* 35 4503–4514. 10.1093/nar/gkm428 17584789 PMC1934995

[B72] Rudt F. Pieler T. (1996). Cytoplasmic retention and nuclear import of 5S ribosomal RNA containing RNPs. *EMBO J.* 15 1383–1391. 10.1002/j.1460-2075.1996.tb00480.x 8635471 PMC450043

[B73] Savada R. P. Bonhamsmith P. C. (2014). Differential transcript accumulation and subcellular localization of *Arabidopsis* ribosomal proteins. *Plant Sci.* 223 134–145. 10.1016/j.plantsci.2014.03.011 24767123

[B74] Sulima S. O. Patchett S. Advani V. M. De Keersmaecker K. Johnson A. W. Dinman J. D. (2014). Bypass of the pre-60S ribosomal quality control as a pathway to oncogenesis. *Proc. Natl. Acad. Sci. U.S.A.* 111 5640–5645. 10.1073/pnas.1400247111 24706786 PMC3992666

[B75] Szick K. Springer M. Bailey-Serres J. (1998). Evolutionary analyses of the 12-kDa acidic ribosomal P-proteins reveal a distinct protein of higher plant ribosomes. *Proc. Natl. Acad. Sci. U.S.A.* 95 2378–2383. 9482893 10.1073/pnas.95.5.2378 PMC19351

[B76] Szick-Miranda K. Zanial A. S. Zanial A. S. Abidayo S. Slater K. L. C. (2010). Analysis of RPS15aE, an isoform of a plant-specific evolutionarily distinct ribosomal protein in *Arabidopsis thaliana*, reveals its potential role as a growth regulator. *Plant Mol. Biol. Report.* 28 239–252. 10.1007/s11105-009-0148-6

[B77] Uechi T. Tanaka T. Kenmochi N. (2001). A complete map of the human ribosomal protein genes: assignment of 80 genes to the cytogenetic map and implications for human disorders. *Genomics* 72 223–230. 10.1006/geno.2000.6470 11401437

[B78] Van de Peer Y. Mizrachi E. Marchal K. (2017). The evolutionary significance of polyploidy. *Nat. Rev. Genet.* 18, 411–424. 10.1038/nrg.2017.26 28502977

[B79] Warner J. R. Mcintosh K. B. (2009). How common are extraribosomal functions of ribosomal proteins? *Mol. Cell* 34 3–11. 10.1016/j.molcel.2009.03.006 19362532 PMC2679180

[B80] Weijers D. Franke-van Dijk M. Vencken R. J. Quint A. Hooykaas P. Offringa R. (2001). An *Arabidopsis* minute-like phenotype caused by a semi-dominant mutation in a ribosomal protein S5 gene. *Development* 128 4289–4299. 11684664 10.1242/dev.128.21.4289

[B81] Weisberg R. A. (2008). Transcription by moonlight: structural basis of an extraribosomal activity of ribosomal protein S10. *Mol. Cell* 32 747–748. 10.1016/j.molcel.2008.12.010 19111651 PMC4161034

[B82] Whittle C. A. Krochko J. E. (2009). Transcript profiling provides evidence of functional divergence and expression networks among ribosomal protein gene paralogs in *Brassica napus*. *Plant Cell* 21 2203–2219. 10.1105/tpc.109.068411 19706795 PMC2751962

[B83] Wilson D. M. I. Deutsch W. A. Kelley M. R. (1994). *Drosophila* ribosomal protein S3 contains an activity that cleaves DNA at apurinic/apyrimidic sites. *J. Biol. Chem.* 269 25359–25364. 10.1016/s0021-9258(18)47256-0 7929231

[B84] Wolfe K. H. Shields D. C. (1997). Molecular evidence for an ancient duplication of the entire yeast genome. *Nature* 387 708–713. 10.1038/42711 9192896

[B85] Wool I. G. (1996). Extraribosomal functions of ribosomal proteins. *Trends Biochem. Sci.* 21 164–165. 10.1016/s0968-0004(96)20011-8 8871397

[B86] Yan H. Chen D. Wang Y. Sun Y. Zhao J. Sun M. (2016). Ribosomal protein L18aB is required for both male gametophyte function and embryo development in *Arabidopsis*. *Sci. Rep.* 6:31195. 10.1038/srep31195 PMC4977502 27502163

[B87] Yang L. Xie C. Li W. Zhang R. J. Jue D. W. Yang Q. (2013). Expression of a wild eggplant ribosomalprotein L13a in potato enhances resistance to *Verticillium dahliae*. *Plant Cell Tiss. Org.* 115 329–340.

[B88] Zettel M. F. Garza L. R. Cass A. M. Myhre R. A. Haizlip L. A. Osadebe S. N. (2003). The budding index of *Saccharomyces cerevisiae* deletion strains identifies genes important for cell cycle progression. *FEMS Microbiol. Lett.* 223 253–258. 10.1016/s0378-1097(03)00384-7 12829295

[B89] Zhang C. Li H. Yuan C. Liu S. Li M. Zhu J. (2020). CKB1 regulates expression of ribosomal protein L10 family gene and plays a role in UV-B response. *Plant Biol. (Stuttg)* 22(Suppl. 1) 143–152. 10.1111/plb.12954 30597713

[B90] Zhao L. J. Cao J. Z. Hu K. X. Wang P. H. Li G. D. He X. D. (2019). RNA-binding protein RPS3 contributes to hepatocarcinogenesis by post-transcriptionally up-regulating SIRT1. *Nucleic Acids Res.* 47 2011–2028. 10.1093/nar/gky1209 30517713 PMC6393244

[B91] Zhou X. Liao W. J. Liao J. M. Liao P. Lu H. (2015). Ribosomal proteins: functions beyond the ribosome. *J. Mol. Cell Biol.* 7 92–104. 10.1093/jmcb/mjv014 25735597 PMC4481666

[B92] Zimmermann R. A. (2003). The double life of ribosomal proteins. *Cell* 115 130–132. 10.1016/s0092-8674(03)00804-3 14567909

[B93] Zorzatto C. Machado J. P. B. Lopes K. V. G. Nascimento K. J. T. Pereira W. A. Brustolini O. J. B. (2015). NIK1-mediated translation suppression functions as a plant antiviral immunity mechanism. *Nature* 520 679–682. 10.1038/nature14171 25707794 PMC4779052

